# Prevalence of vero toxic *Escherichia coli* in fecal samples of domestic as well as wild ruminants in Mathura districts and Kanpur zoo

**DOI:** 10.14202/vetworld.2016.71-74

**Published:** 2016-01-21

**Authors:** Raghavendra Prasad Mishra, Udit Jain, Basanti Bist, Amit Kumar Verma, Ashok Kumar

**Affiliations:** 1 Department of Veterinary Public Health, College of Veterinary Sciences and Animal Husbandry, Pandit Deen Dayal Upadhayay Pashu Chikitsa Vigyan Vishvidhyalaya Ewam Go-Anusandhan Sansthan, Mathura - 281 001, Uttar Pradesh, India; 2 Department of Veterinary Epidemiology and Preventive Medicine, College of Veterinary Sciences and Animal Husbandry, Pandit Deen Dayal Upadhayay Pashu Chikitsa Vigyan Vishvidhyalaya Ewam Go-Anusandhan Sansthan, Mathura - 281 001, Uttar Pradesh, India; 3 Division of Veterinary Public Health, Indian Veterinary Research Institute, Izatnagar, Bareilly, Uttar Pradesh, India

**Keywords:** domestic and wild ruminants, feces, prevalence, polymerase chain reaction, verotoxic *Escherichia coli*

## Abstract

**Aim:**

The present study was planned to reveal the prevalence of verocytotoxigenic *Escherichia coli* (VTEC) in fecal samples of domestic and wild ruminants in Mathura district and Kanpur zoo.

**Materials and Methods:**

A total of 240 fecal samples comprising 60 each of cattle, buffalo, sheep and deer from Mathura districts and Kanpur zoo were screened for the presence of *E. coli* and VTEC genes positive by polymerase chain reaction (PCR).

**Result:**

Out of 240 fecal samples, 212 *E. coli* strains were obtained. All the *E. coli* isolates were screened by PCR to detect virulence genes *stx_1_*, *stx_2_*, *eaeA* and *hlyA*. Of these, 25 isolates were identified as VTEC. The prevalence of VTEC in cattle, buffalo, sheep and deer was found 13.4% (8/60), 13.4% (8/60), 6.67% (4/60) and 8.33% (5/60), respectively.

**Conclusion:**

*stx_1_*, *stx_2_*, *eaeA* and *hlyA* genes were prevalent in VTEC isolates from feces of cattle, buffalo, sheep and deer population of Mathura districts and Kanpur zoo. The presence of VTEC isolates in this region may pose a threat to public health.

## Introduction

Diarrhea is one of the most common multifactorial diseases of man and animals mainly caused by *Escherichia coli* [1]. *E. coli* is the most common observed gastrointestinal flora of animals and environmental contaminant considered as important foodborne pathogen causing serious complications in man and animals [2-5].

Verocytotoxigenic *E. coli* (VTEC) was the first identified as a distinct group of *E. coli* named as VTEC, which had the ability to produce toxins with profound and irreversible effect on vero cells. VTEC is also termed as shiga-like toxin producing *E. coli* (SLTEC) or shiga toxin producing *E. coli* or STEC. Acronym STEC is derived from the fact that the toxins are shiga like that is similar to those produced by *Shigella dysenteriae* Type 1 [6].

The enterohemorragic *E. coli* belong to the VTEC. VTEC always do not induce clinical signs and are not enterohemorrhagic until addition virulence factor are present like enterohemolysin and adherence factors (intimin). The adherence factor(s) enables the organism to attach to and colonize intestinal mucosal cells [7]. Among VTEC, serotype O157:H7 has been closely associated with the sporadic and clinical outbreaks of hemorrhagic colitis (HC), hemolytic uremic syndrome (HUS) and thrombotic thrombocytopenic purpura (TTP) in human beings [8-10]. Healthy domestic ruminants are recognised as the main natural reservoir of STEC and large game animal may be healthy carriers of STEC [11,12].

Keeping in view the importance of this organism, the present study was planned to reveal the prevalence of VTEC in fecal samples of domestic and wild ruminants in Mathura district and Kanpur zoo.

## Materials and Methods

### Ethical approval

This work does not require ethical approval because we have collected fecal samples of animals after defecation.

### Sampling and isolation of E. coli

A total of 240 samples of feces (180 domestic ruminants 60 wild ruminants) were collected from Mathura district and Kanpur zoo. The samples were collected aseptically in UV sterile polythene bags (Fisher Scientific, UK) and immediately transported to the laboratory under chilled conditions for microbiological analysis. For primary isolation of *E. coli* (VTEC), 10 g of fecal sample were enriched in 90 ml modified trypticase soya broth (mTSB) (Himedia, Mumbai) containing acriflavine (10 mg/ml) to reduce the growth of Gram-positive organism. The method used for collection of materials, and isolation and identification techniques were performed as suggested by the World organization for Animal Health [13]. These samples were incubated at 37°C for 6 h. MacConkey’s agar was used as differential media while eosin methylene blue agar (Hi-Media, Mumbai) was used as selective media. Suspected *E. coli* strains were subjected to morphological, cultural and biochemical characterization as per standard methods [14]. A statistical analysis was done as per the standard method [15].

### Molecular characterization

Multiplex polymerase chain reaction (pcr) was used for detection of virulent genes (*stx_1_, stx_2_*, *eaeA*, and *hlyA*) of VTEC. All the *E. coli* isolates were subjected to genomic DNA isolation. The bacterial growth in mTSB broth (HiMedia, Mumbai) was centrifuged at 3000 rpm for 15 min to make the pellet of bacterial cells. These cells were washed twice with phosphate-buffered saline (pH 7.4) to remove any impurity of broth media. Bacterial DNA was extracted by using DNA extraction kit (Genei, Bangalore) as per the manufacturer’s protocol. For the PCR reaction, PCR Master Mix solution (Genei, Bangalore) was used. DNA amplification targeted to virulent genes (*stx_1_*, *stx_2_*, *eaeA* and *hlyA*) of VTEC was performed using primers on 3 µl of DNA sample in 25 µl reaction mixture [16]. After an initial denaturation step at 95°C for 4 min, 30 amplification cycles were performed, each consisting of 2 min at 94°C, 2 min at 65°C, and 1.5 min at 72°C and followed by a final extension step at 72°C for 2.5 min. After the amplification, amplicons were separated in 1.5% gel in tris acetate EDTA (TAE) buffer at 60 volt for 80 min, stained with 0.5% ethidium bromide solution and visualized under ultraviolet light.

## Results and Discussion

Out of 240 fecal samples, a total of 212 *E. coli* strains were obtained (Table-1). All the strains of *E. coli* were screened to detect the presence of VTEC genes using PCR. An overall prevalence of VTEC in ruminants (both wild and domestic) was found to be 10.42% (25/240). The highest prevalence of VTEC was reported in cattle and buffalo 13.4% (8/60) in each followed by sheep 6.67% (4/60) and deer 08.33% (5/60). In cattle, 2 VTEC were found to be positive for *stx_1_* gene (180 bp) and 6 VTEC for *stx*_1_ and s*tx_2_* (180 bp and 255 bp). In buffalo, 3 VTEC were found positive for *stx_1_* gene and 5 was positive for *stx_1_* and *stx_2_*. In sheep, out of 4 VTEC, only one VTEC was having *stx_1_* gene and 3 VTEC isolates having *stx_1_* and *stx_2_* both. In wild ruminants (deer), out of 5 VTEC, only one was found to be positive for *stx_1_* gene, one isolates was found to be positive for *stx_2_* with *hlyA* (534 bp), one *stx_1_* with *eaeA* (384 bp) and two VTEC have *stx_2_*, *eaeA* and *hlyA* genes. Two *E. coli* strains were found having *eaeA* with *hlyA* genes, i.e., lacking *stx* gene and they may be enteropathogenic.

**Table-1:** Details of sample collection and prevalence of *E. coli* and VTEC in fecal sample.

Sources*	Place of collection	Number of sample collected	Percentage of *E. coli*	No. of VTEC isolates	Percentage of VTEC
Cattle	DDD farm DUVASU, Mathura	35	85.7 (30/35)	7	20
	Gaushalas of Vrindavan	15	93.4 (14/15)	1	6.67
	TVCC Kothari	10	90 (9/10)	0	0
Buffalo	DDD farm DUVASU, Mathura	40	95.6 (38/40)	8	20
	TVCC Kothari	20	90 (18/20)	0	0
Sheep	Sheep farm DUVASU, Mathura	30	86.7 (26/30)	2	6.67
	Sheep farm, farah	20	80 (16/20)	1	5
	Aurangabad	10	70 (7/10)	1	10
Deer	Kanpur Zoo	30	86.7 (26/30)	2	6.66
	Ramanreti	30	93.4 (28/30)	3	10
Total		240	88.5 (212/240)	25	10.41 (25/240)

p<0.05, VTEC=Verocytotoxigenic *Escherichia coli, E. coli=Escherichia coli*

In the previous study, the prevalence of VTEC in sheep, cattle and buffalo were reported as 4.81% [17], 7.4% [18] and 8.9% [19], respectively. However, the prevalence of VTEC in higher level was reported by previous workers as the prevalence of VTEC was 16.66% [20], 18% [21] and 18.47% [22]. In contrast, investigations have shown a higher detection rate of 46% [23] in fecal samples of cattle and buffalo. Lower isolation rate, i.e. 9% [24]. Isolation rate as low as 1.0% has also been reported [25]. In deer previously reported, the prevalence of VTEC 9.3% [26], 16.2% [27] and16.5% [28], which likely similar as present finding. These hazardous strains of *E. coli* have been given immense attention due to their involvement in serious illnesses like HC (bloody diarrhea, HC), HUS and TTP in human [29-31]. The low infective dose, unusual acid tolerance and close association with ruminants have made VTEC a serious global zoonotic problem of great public health significance. VTEC can be present in the intestinal tract of a wide range of domestic and wild animals and ruminants (sheep, goats, cattle, buffalo and deer) [32-36], especially cattle and buffalo are considered as a major reservoir for VTEC [37,38].

## Conclusion

The presence of VTEC in feces causes fecal contamination of water, and also contaminates other food sources, thus depicts a dangerous picture regarding human and animal health safety because water is essential for the survival of every living being. Constant monitoring and surveillances program to keep a record of the prevalence from time to time is needed, and proper hygienic measure may reduce the chance of infection.

## Authors’ Contributions

UJ, BB and AKV designed and planned this research work. RPM collected the samples and executed the isolation and biochemical work. UJ monitored the isolation, biochemical characterization. RPM and UJ was involved in the molecular characterization experiment. Manuscript was drafted and revised by AK, BB & RPM under the guidance of UJ. All authors read and approved the final manuscript.
